# Attitude towards assisted reproductive technology: acceptance of donors eggs, sperms, and embryos as treatment of human infertility: a systematic review and meta-analysis

**DOI:** 10.1186/s12978-024-01741-0

**Published:** 2024-01-23

**Authors:** Dereje Bayissa Demissei, Tolesa Diriba Biratu, Eriste Nigussa Gamshe, Ababe Tamirat Deressa

**Affiliations:** 1 School of Nursing, Saint Paul’s Hospital Millennium Medical College, Addis Ababa, Ethiopia; 2 School of Public Health, St. Paul’s Hospital Millennium Medical College, Addis Ababa, Ethiopia

**Keywords:** Attitude, Donors’ eggs, Embryos, Sperm, Acceptance, Infertility treatment

## Abstract

**Introduction:**

Assisted Reproductive Technology utilizes human sperm, eggs, or embryos in vitro to produce pregnancy. However, there is no evidence of the acceptance of these technologies by the community.

**Objective:**

This study aimed to determine the pooled prevalence of positive attitudes toward the acceptance of donor eggs, embryos, and sperm.

**Methods:**

The protocol was registered in PROSPERO (number: CRD42022348036). The Condition, Context and Population (CoCoPop) protocol of the systematic review was used to address the relevant questions regarding the objective of the study. Data were extracted into Excel and pooled estimates were calculated using STATA Version 16.

**Results:**

The pooled prevalence of positive attitudes toward accepting donor eggs, embryos, and sperms was 38.63%, 33.20%, and 31.34%, respectively. Subgroup analysis revealed that the pooled prevalence of positive attitudes toward accepting donor eggs was high in non-Asian countries (47.78%) and among infertile men (38.60%). Similarly, the pooled prevalence of positive attitudes toward accepting donor eggs was high in non-Asian countries (47.78%) and among infertile men (28.67%). However, the pooled prevalence of positive attitudes toward accepting donor sperm was high in non-Asian countries (37.6%) and among infertile women (28.19%).

**Conclusion:**

The pooled estimate of the prevalence of positive attitudes toward accepting donor eggs was higher than the prevalence of positive attitudes toward accepting donor embryos and sperm. Infertile men and non-Asian countries have a higher prevalence of positive attitudes toward accepting eggs and embryos, whereas non-Asian countries and infertile women present a higher prevalence of positive attitudes toward accepting donor sperm. Therefore, regulatory bodies and policymakers should modify their rules and regulations to ensure the availability of minimum standards for the ethical and safe practice of donor conception as a treatment for infertility at national and international levels.

**Supplementary Information:**

The online version contains supplementary material available at 10.1186/s12978-024-01741-0.

## Plain language summary

Assisted Reproductive Technology (ART) utilizes human sperm, eggs, or embryos in vitro to induce pregnancy; however, there is no evidence of community acceptance of these technologies. A systematic review and meta-analysis found that 38.63% of infertile couples had positive attitudes toward donor eggs, while 33.20% and 31.34% had negative attitudes. Females are more amenable to accepting donor gametes, embryos, or eggs than males, and females are more amenable to accepting donor eggs than donor sperm. To improve attitudes toward donor conception, infertile couples must understand the medical and obstetric risks associated with donor-assisted conception. This review recommends strengthening counseling for infertile couples and offering support to those with negative attitudes toward donor conception. Regulatory bodies and policymakers should consider the needs of infertile couples and modify their rules to ensure minimum standards for ethical and safe practices of donor conception as a treatment for infertility.

**Supplementary Information:**

The online version contains supplementary material available at 10.1186/s12978-024-01741-0.

## Introduction

Assisted Reproductive Technology (ART) involves all treatments and procedures with the aim of inducing pregnancy using human sperm, eggs, or embryos in vitro. It mainly includes in vitro fertilization and embryo transfer, gamete intrafallopian transfer, zygote intrafallopian transfer, tubal embryo transfer, gamete and embryo cryopreservation, oocyte and embryo donation, and gestational surrogacy. However, assisted insemination (artificial insemination) using sperm from either a woman’s partner or a sperm donor is not included in assisting reproductive technology [1].

An embryo recipient cycle is defined as an ART cycle in which a woman’s uterus is prepared to receive one or more cleavage-stage embryos or blastocysts resulting from gametes that do not originate from their male partner, if present. Furthermore, receiving eggs/oocytes is an ART procedure in which a woman receives oocytes from a donor, or her partner if they are in a same-sex relationship, to be used for reproductive purposes. In contrast, receiving sperm is a technology in which a woman receives spermatozoa from a person who is not a sexually intimate partner [2].

Global evidence stated that utilization of all ART cycles was lowest in Latin America (16.0%) and highest in Japan and in Australia and New Zealand, encompassing 82.6% and 76.3%, respectively [3]. However, community approval for the use of in vitro fertilization (IVF) for the treatment of infertility has risen significantly in Australia over the past 20 years. For instance, Support for IVF to support infertile married couples increased from 77% in 1981 to 86% in 2001. Again, Medicare funding increased from 70% in 1981 to 79% in 2000 [4].

Provision of awareness creation over a longer period and political affiliation were significantly related to positive attitudes toward ART utilization in the USA [5]. In Southeastern Michigan, couples favorably viewed the interventions of Assisted Reproductive Technologies. Low acceptance of in vitro fertilization (IVF) has been reported as a possible cause of new interventions or high cost, which is not covered by third parties [6]. The couples who participated in a study from Canada were willing to use ART if they faced infertility challenges. However, women are less willing to use donated eggs and embryos, gestational surrogacy, and fertility preservation than are men [7].

A pilot study from Germany reported that assisted reproductive technology is socially acceptable overall, and the majority of respondents stated that they would utilize it when needed. The native group showed the lowest acceptance rates compared with migrants from Poland and Turkey [8]. In the same country, religious affiliation was associated with an open attitude toward ART utilization. In this study, Christian follower women had a less favorable attitude toward using assisted reproductive technologies than Muslim women [9].

Although Assisted Reproductive Technology is often reported to be incorrectly considered as a treatment for age-related infertility in Spain and Israel [10], the largest share (95%) of the participants undergoing reproductive donation treatment in Spain perceived undergoing gamete donation as an acceptable decision [11]. In Ireland, patients with a history of infertility for long periods are more likely to accept oocyte donation than those who have lived with infertility for a shorter duration [12]. Likewise, older respondents from Japan are more likely to express positive attitudes toward egg donation by traveling overseas [13].

A study conducted on the community’s attitude toward Assisted Reproductive Technology in Iran stated that they did not support all types of assisted reproduction. The most widely accepted method for infertility treatment is in vitro fertilization (IVF) (using the husband’s sperm and the wife’s eggs) [14]. In the same country, a study showed that infertile couples had a positive attitude toward Assisted Reproductive Technology. A couple’s attitude, their family’s attitude, and applied knowledge of ART influence decision-making and acceptance of the methods [15].

A study conducted in an infertility center in India reported different levels of acceptance of ART among male and female partners. Accordingly, 19.9%, 19.5%, and 15.7% of female partners agreed with using donor eggs, donor sperm, and donor embryos, respectively. On the other hand, 44.1%, 15.2%, and 23.7% of the male partners agreed with using donor eggs, donor sperm, and donor embryos, respectively [16]. It has been reported in Egypt that 85% of infertile couples have positive attitudes toward ART. The level of knowledge did not show significant statistical differences based on couples’ sociodemographic characteristics and attitudes toward reproductive methods [17]. The majority of study participants in Urban Lagos, Nigeria, had poor knowledge but a positive attitude toward ART utilization; thus, they pointed out the need to create awareness and concern about reducing the cost of using these methods [18]. In Northern Nigeria, 18%, 29%, and 7.3% of the infertile women who participated in the study had favorable attitudes toward use of donor sperm, donor oocytes, and donor zygotes, respectively [19].

Artificial intelligence (AI) is increasingly being utilized in medicine to improve infertility diagnosis and ART outcomes, particularly in cases of recurrent ART failure. AI applications include ultrasound monitoring, endometrial receptivity, embryo selection, and post-implantation embryo development prediction. Oocyte morphology assessment is crucial for successful fertilization rates and fertility preservation. AI has also been used in male infertility assessment, with computerized semen analysis systems already in use. Advances in AI in idiopathic infertility are also being made [20]. AI is revolutionizing reproductive medicine by improving treatment options, planning procedures, and predicting clinical outcomes. It will reduce treatment costs and improve ART success rates. However, incorporating AI in everyday practice requires careful consideration of risk-assessment systems and care delivery. While AI will not replace human presence, it will aid in decision-making, saving time in infertility treatment. However, careful drafting of ethical frameworks is crucial [20].

Cell-free fetal DNA (cffDNA) analysis is a non-invasive prenatal diagnostic test used to screen chromosomic or monogenic pathologies in a fetus. A critical appraisal of 45 studies on this technique has highlighted its well-established diagnostic value. The review discusses the non-invasive prenatal diagnosis using fetal cell-free DNA, highlighting its importance in the first trimester of pregnancy. It emphasizes the need for scientific community to provide accurate diagnostic definitions while maintaining clinical, ethical, and legal viability, considering the best standards for this testing technique [21].

A systematic review of ART procedure results in conflicting results regarding the risk of coronary heart disease (CHDs) in pregnancies. A study analyzing 24 studies found a 3% pooled incidence of congenital heart disease in ART pregnancies, with a 1% decrease for major CHDs. ART pregnancies have an increased risk of minor CHDs, but insufficient evidence for major CHDs [22].

International guidelines are crucial for the implementation of counselling in oncofertility, a fertility preservation procedure. These guidelines aim to ensure that patients have access to fertility preservation procedures, while also adhering to ethical and legal standards. Counselling should be rooted in collaboration between oncologists, reproductive endocrinologists, mental health counsellors, and clinical researchers, ensuring that the provision of oncofertility services upholds individual autonomy and does not pose a risk to the children conceived or others [23].

## Methods

### Protocol and registration

The protocol was registered (number: CRD42022348036) in the International Prospective Register of Systematic Reviews (PROSPERO).

### Eligibility criteria and search strategies

We followed the Preferred Reporting Items for Systematic Reviews and Meta-analysis (PRISMA) guideline [24] to prepare the whole document. National surveys and published and unpublished articles were retrieved from various databases. Additionally, the reference lists of the included articles were crosschecked to identify articles that were not assessed in the search strings. A comprehensive search of research literature published on PubMed, CINAHL (EBSCO host), Global Health (CABI), Medline (EBSCO host), and other sources (Google Scholar and Google) that reported “Attitude” OR “Perception” OR “Acceptance” “donor” AND “Eggs” OR “Embryo” OR “Sperm” was performed in this systematic review and meta-analysis.

### Inclusion and exclusion criteria

The question format for this meta-analysis was as follows: Condition, Context, Population (CoCoPop) [25]. The CoCoPop framework is used for reviews addressing a question related to the prevalence of positive attitudes toward acceptance of ART among infertile couples: (a) condition (acceptance or recipient of donor sperm, embryo, egg, oocyte, or ova); (b) context (global, regional, and national), study design (cohort studies, cross-sectional studies, epidemiology, observational studies), and study setting (community-based surveys, health institutions, web-based surveys); and (c) population (infertile couples). Data from each study were verified for eligibility using the study area, study set-up, assessment methods, study designs, titles, abstracts, and full texts. Eventually, studies written in English reporting the magnitude of positive attitudes toward the acceptance of donated sperm, embryos, and eggs and their acceptance among infertile couples worldwide were included.

Exclusion criteria: Similar patients were enrolled in different articles, commentaries, editorials, case reports, letters, family-based studies, and short communication. However, studies with incomplete or unclear acceptance of eggs/ovum/sperm or embryo service operational definitions and those without full text were excluded. Letters to the editors, conference proceedings, and qualitative studies were also excluded. EndNote X8 reference manager was used to manage the articles.

### Search strategies and selection process of studies

The appropriateness of the key terms was checked prior to conducting searches in each database. An example of a search string in PubMed is as follows: ((((Acceptance of Donor Eggs) OR (“Sperms”[Mesh])) OR (Donor Sperms)) AND (“Donor Embryos”[Mesh]) (“Egg Recipient”[Mesh]) AND ((2012 OR (“Sperm Injections, Intracytoplasmic”[Mesh])) AND (“Attitude to Health”[Mesh])) OR (“Intention”[Mesh])) AND (humans[Filter]) (“Infertility”[MeSH]) (data[Filter]))). The Boolean operators AND OR were used accordingly (see Additional file 1: Appendix S1).

### Data extraction procedures

Data were extracted by four independent investigators (Dereje Bayissa Demissie and Tolessa Diriba Biratu), and the third and fourth authors (Ababe Tamirat Deressa and Eriste Nigussa Nugusa) resolved any inconsistencies.

Inter-rater agreement was computed by one of the authors (DBD) before inclusion in this study was decided. Inter-rater agreement was computed using Cohen’s kappa coefficient (κ). The findings revealed a substantial agreement [26] between the two raters (κ = 0.62, p < 0.01).

The extracted data included the first author’s name, publication year, continent, study country, study period, study design, sample size, prevalence of acceptance of donor eggs/ovum/sperm or embryos, and number of infertile couples. The data were summarized using a Microsoft Excel 2016 spreadsheet (Additional file 1: Appendix S1).

### Quality assessment

The quality of the study was assessed using the Joanna Briggs Institute (JBI) critical appraisal tools [27], and the results were graded as low, medium, or high if the quality score was < 60%, 60–80%, or > 80%, respectively [28].

### Publication bias and heterogeneity

Funnel plots and Egger’s regression test were used to measure publication bias at a 5% significance level [29]. In addition, heterogeneities among the studies used to compute the pooled estimates in this meta-analysis were explored using forest plots, I^2^ tests, and Cochrane Q statistics [30]. I^2^ values of 25%, 50%, and 75% were interpreted as low, medium, and high heterogeneity, respectively [31]. In the current meta-analysis, significant heterogeneity was considered when the I^2^ value was ≥ 50%, with *a p-value* < *0.05*. We inspected the funnel plot and conducted Egger’s regression tests to assess publication bias [28]. A trim-and-fill analysis was conducted to adjust for publication bias [32]. The possible sources of significant heterogeneity were addressed through subgroup and sensitivity analyses.

### Outcome and summary measures

The primary outcome of this study was the pooled prevalence of positive attitudes toward the acceptance of donor eggs, sperm, or embryos. The CoCoPop framework was used for reviews that addressed questions relevant to the prevalence of positive attitudes toward egg acceptance.

Co- Condition or problem, Co-Context the question is set and Pop- Population being examined.

CoCoPop Example: What is the prevalence of positive attitude acceptance of donors Eggs, sperms, and Embryos (Condition) Globally (Context) of infertile couples (Population)?

CoCoPop represents Condition, Context, Population [25]. Condition refers to the use of donor eggs and embryos in infertility cases in which the recipient’s eggs are either absent, unavailable, or inappropriate for in vitro fertilization (IVF) and embryo transfer (ET) programs. Human pregnancies and live births have recently been reported following the donation of embryos fertilized both in vitro and in vivo [33].

Context indicates the global, regional, and national pooled prevalence acceptance of donor eggs, donor sperm, or donor embryos among infertility cases.

The primary outcome of this study was to estimate the global acceptance of donor eggs, sperm, and embryos among infertile couples. The pooled prevalence of acceptance of donor eggs, sperm, or embryos was computed globally, for infertile couples, and for Asian and non-Asian countries.

### Data synthesis and statistical analysis

**Fig. 1:**
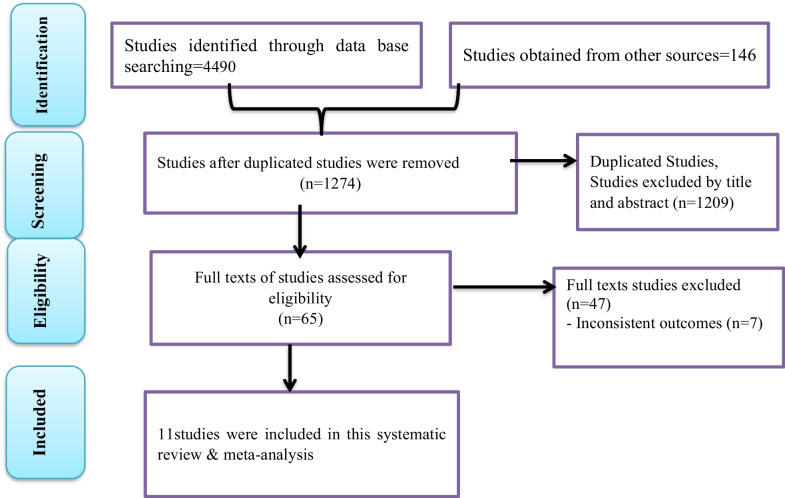
PRISMA flow chart showing the study selection process

Pooled estimates were calculated using STATA Version 16 (STATA Corporation, College Station, Texas, USA). Both random- and fixed-impact methods were used to measure the pooled estimates. The pooled estimates were computed using “metaprop” with sample size as a weight (wgt) variable with 95% CIs. Pooled estimates were computed using random-effects models and weighted using the inverse variance method in the presence of high heterogeneity among studies. Subgroup analyses were performed using different parameters (continent and study country). We verified the appropriateness of each datum prior to analysis. Forest plots, summary tables, and text are used to present the findings of this study (Fig. 1).

### Study characteristics

Eleven studies with a total sample size of 6582 were included in the final analysis [7, 12, 13, 16, 34–40].

All studies were cross-sectional studies, and critical appraisal of the cross-sectional studies revealed that approximately 95% of studies scored high quality and only 5% scored medium quality assessment (Tables 1 and 2).

**Table 1:** Attitude toward sperm, embryo, eggs/oocyte/ovum acceptance. Detailed description of included studies for computing the prevalence of attitudes, acceptance of donor eggs, donor sperm, or donor embryos, egg recipient and sperm recipient among infertile couples globally

Author, year	Study population	Study design	Study area/country	Sample size	Age/mean/median	Acceptance of donor eggs/oocyte/ovum (%)	Acceptance of donor sperm (%)	Acceptance of donor embryos (%)	Overall quality result
(Straehl et al., 2017) [38]	Infertile women	Hospital-based Cross-sectional	Brazil	69	34.5	58.0			High
(Daniluk and Koert, 2012) [7]	Infertile women	Community-based Cross-sectional	Canada	2000		24.7	31.2	20.3	High
(Daniluk and Koert, 2012) [7]	Infertile men	Community-based Cross-sectional	Canada	599		34	25.8	29.1	High
(Hibino et al., 2013) [13]	Infertile patients	Clinic-based cross-sectional	Japan	2007	36.3	10	10	10	Medium
(Banerjee and Singla, 2022) [16]	Infertile women	Clinic-based cross-sectional	India	594		19.9	19.5	15.7	High
(Banerjee and Singla, 2022) [16]	Infertile men	Clinic-based cross-sectional	India	594		44.1	15.2	23.7	High
(Afshani et al., 2016) [15]	Infertile men	Clinic-based cross-sectional	Iran	86	31	37.14	37.14	37.14	High
(Afshani et al., 2016) [15]	Infertile women	Clinic based cross-sectional	Iran	98	31	36.12	36.12	36.12	High
(Ahmadi and Bamdad, 2017) [14]	Public-women	Community-based Cross-sectional	Iran	276	28.8	35.4	29.3		Medium
(Ahmadi and Bamdad, 2017) [14]	Public-men	Community-based Cross-sectional	Iran	129	33.9	32.8	15.6		High
(Baccino et al., 2014) [40]	Infertile couples	Hospital-based Cross-sectional	FivMadrid, Madrid, Spain	130		94	94	94	High

**Table 2:** Joanna Briggs Institute’s Quality Assessment tool to include studies in a Systematic Reviews and Meta-Analysis

Author, year	Q1				Q2				Q3				Q4				Q5				Q6				Q7				Q8				Q9		Overall quality result
	Y	N	U	NA	Y	N	U	NA	Y	N	U	NA	Y	N	U	NA	Y	N	U	NA	Y	N	U	NA	Y	N	U	NA	Y	N	U	NA	Y	N	
(Straehl et al., 2017) [38]	√				√				√				√							√				√	√				√				√		High
(Daniluk and Koert, 2012) [7]	√				√				√				√							√				√	√				√				√		High
(Daniluk and Koert, 2012) [7]	√				√				√						√					√				√	√				√				√		High
(Hibino et al., 2013) [13]	√				√				√				√				√				√				√				√				√		Medium
(Banerjee and Singla, 2022) [16]	√				√				√				√				√				√				√				√				√		High
(Banerjee and Singla, 2022) [16]	√				√				√				√							√				√	√				√				√		High
(Afshani et al., 2016) [15]	√				√				√				√				√				√				√				√				√		High
(Afshani et al., 2016) [15]	√				√				√				√							√				√	√				√				√		High
(Ahmadi and Bamdad, 2017) [14]	√				√				√				√							√				√	√				√				√		Medium
(Ahmadi and Bamdad, 2017) [14]	√				√				√				√							√				√	√				√				√		High
(Baccino et al., 2014) [40]	√				√				√				√				√				√				√				√				√		High
(Straehl et al., 2017) [38]	√				√				√				√				√				√				√				√				√		High
(Daniluk and Koert, 2012) [7]	√				√				√				√				√				√				√				√				√		High
(Daniluk and Koert, 2012) [7]	√				√				√				√				√				√				√				√				√		Medium
(Hibino et al., 2013) [13]	√				√				√				√							√				√	√				√				√		High
(Banerjee and Singla, 2022) [16]	√				√				√				√							√	√				√				√				√		High
(Banerjee and Singla, 2022) [16]	√				√				√				√				√				√				√				√				√		High
(Afshani et al., 2016) [15]	√				√				√				√							√				√	√				√				√		High
(Afshani et al., 2016) [15]	√				√				√				√							√				√	√				√				√		Medium
(Ahmadi and Bamdad, 2017) [14]	√				√				√				√							√				√	√				√				√		High
(Ahmadi and Bamdad, 2017) [14]	√				√				√				√							√				√	√				√				√		High
(Baccino et al., 2014) [40]	√				√				√						√					√				√	√				√				√		High

*Y* yes, *N* no, *U* unclear, *NA* not applicable, *< 60%* low, *60–80%* medium, *> 80%* high quality

## Results

### Selection of studies

In the initial search, 4490 studies were obtained from databases and gray literature sources. A total of 1209 studies were excluded due to duplication. Then, 3216 studies were screened using titles and abstracts, and 1274 were removed. Finally, the full texts of 65 studies were assessed for eligibility. Of these 65 studies, 54 were excluded due to inconsistent results. Ultimately, 11 eligible studies were included in the final analysis of the current systematic review and meta-analysis [7, 12, 13, 16, 34–40]. Of the 11 studies, all 11 included egg acceptance, 10 included sperm acceptance, and 8 included embryo acceptance individual prevalence, respectively.

### Global pooled prevalence of positive attitudes toward donated egg acceptance

Eleven studies with a total sample size of 6582 were included in the final analysis; the sample size ranged from 69 to 2007 [13, 38].

The range of positive attitudes toward egg acceptance was reported from 10% in Japan [13] to 94% in Spain [38].

Of the 11 studies, all 11 included egg acceptance, 10 sperm acceptance, and 8 included embryo acceptance individual prevalence, respectively.

**Fig. 2:**
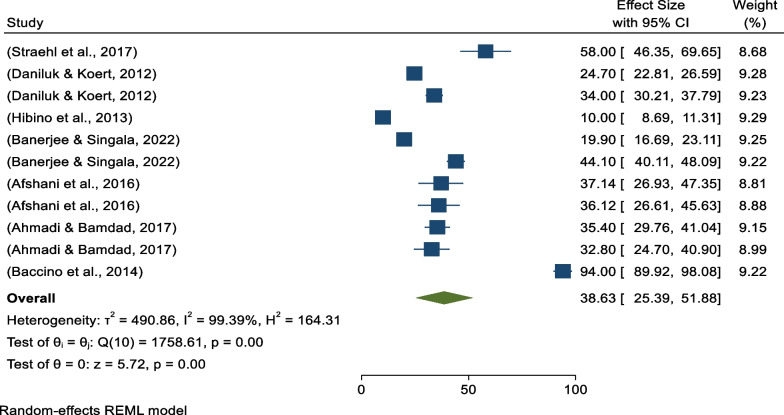
Forest plot showing the global pooled prevalence of positive attitudes toward egg acceptance per 100 among infertile couples 2022

Based on the random-effects model, the global pooled positive attitudes toward egg acceptance was 38.63% (95% CI 25.39% − 51.88%) per 100 among infertile couples (Fig. 2). There was high heterogeneity among studies (*I*^*2*^ = 99.39%, *p* = 0.001).

### Subgroup analysis of positive attitudes toward egg acceptance

**Fig. 3:**
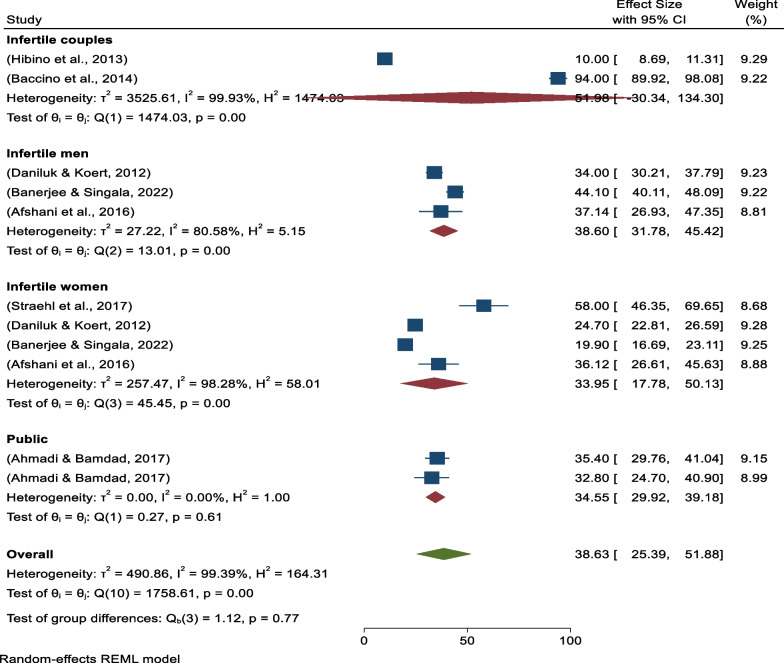
Forest plot showing study participant-based sub-group analysis for pooled prevalence of positive attitudes toward donated egg acceptance

First, a study participant-based subgroup analysis was undertaken, which revealed three studies conducted on infertile men with pooled prevalence of 38.60% (95% CI 31.78%–45.42) (*I*^*2*^ = 80.58%, *p* = 0.00), four studies conducted on infertile women with pooled prevalence of 33.95% (95% CI 17.78–50.13) (*I*^*2*^ = 98.28%, *p* = 0.00), and two studies conducted in the community (public) with pooled prevalence 34.55% (95% CI 29.92–39.18) (*I*^*2*^ = 0.00%, *p* = 0.61) (Fig. 3).

**Fig. 4:**
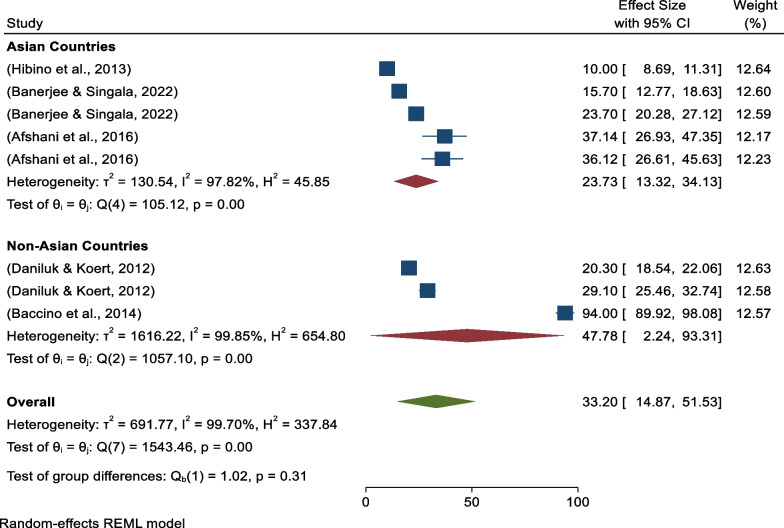
Forest plot showing continental sub-group analysis for pooled prevalence of positive attitudes toward donated egg acceptance

Next, continental subgroup analysis revealed five studies were conducted in Asia with pooled prevalence of 23.73% (95%CI 13.32–34.13) (*I*^*2*^ = 97.82%, *p* = 0.00), and three studies were conducted in non-Asian countries with pooled prevalence of positive attitudes toward donated egg acceptance of 47.78% (95%CI 2.24–93.31) (*I*^*2*^ = 99.85%, *p* = 0.00) (Fig. 4).

**Fig. 5:**
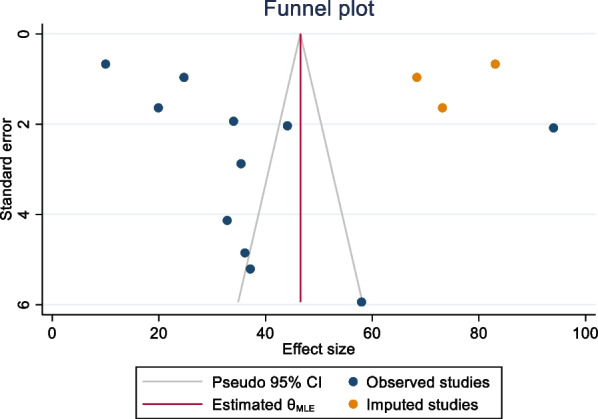
Funnel plot showing the trim-and-fill analysis on global pooled prevalence of positive attitudes toward egg acceptance

Publication bias was assessed using funnel plots and objectively verified using Egger’s regression test. The funnel plot appeared asymmetric, although Egger’s regression test (p = **0.3843**) did not confirm the asymmetry of the funnel plot (Fig. 5). Finally, the funnel plots appear asymmetrically pin-pointed to the right for global pooled prevalence of positive attitudes toward donated egg acceptance and warrant the acknowledgment of possible publication bias within the article, which represents the current body of literature.

### Global pooled prevalence of positive attitudes toward donated embryo acceptance

Eight studies with a sample size of 6,108 were included in the final analysis, and the sample sizes of the individual studies ranged from 86 to 2007 [19, 22]. The range of positive attitudes toward embryo acceptance was reported from 10% in Japan [13] to 94% in Spain [38].

**Fig. 6:**
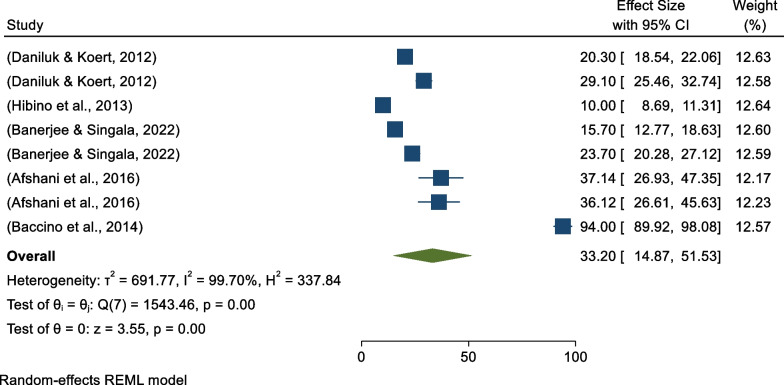
Forest plot showing the global pooled prevalence of positive attitudes toward embryo acceptance per 100 among infertile couples 2022

Based on the random-effects model, the global pooled prevalence of positive attitudes toward embryo acceptance was 33.20% (95% CI 25.39%–51.88%) (Fig. 6). There was high heterogeneity among studies (*I*^*2*^ = 99.70%, *p* = 0.00).

### Subgroup analysis of positive attitudes toward embryo acceptance

**Fig. 7:**
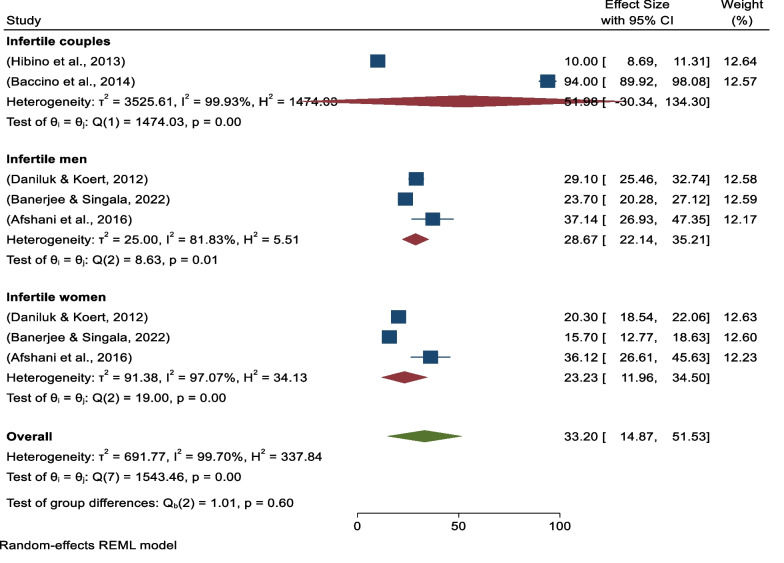
Forest plot showing study participant-based sub-group analysis for pooled prevalence of positive attitudes toward donated embryo acceptance

Based on the category of study participants, subgroup analysis revealed that two studies were conducted on infertile couples at the public level with pooled prevalence of 51.98% (95% CI − 30.34–134.30) (*I*^*2*^ = 99.93%, *p* = 0.00), three studies were conducted on infertile men with pooled prevalence of 28.67% (95% CI 22.14–35.21) (*I*^*2*^ = 81.83%, *p* = 0.01), and three studies were conducted on infertile women with pooled prevalence of positive attitudes toward embryo acceptance of 23.23% (95% CI 11.96–34.50%) (*I*^*2*^ = 97.07%, *p* = 0.00) (Fig. 7).

**Fig. 8:**
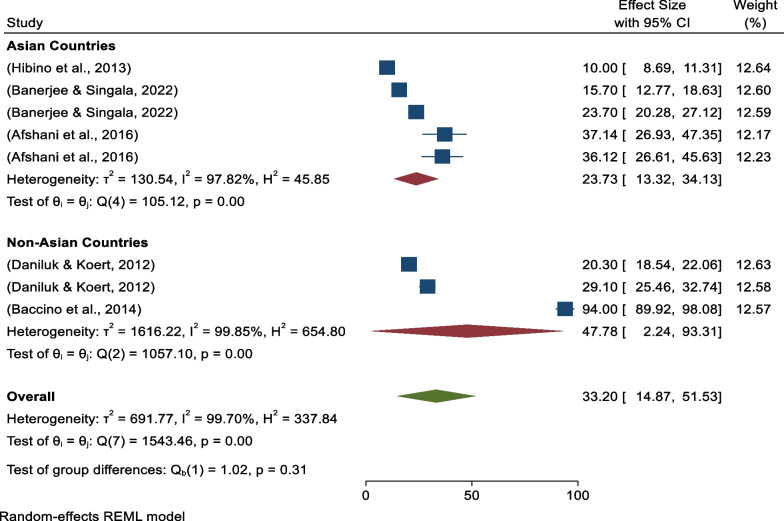
Forest plot showing continental sub-group analysis for pooled prevalence of positive attitudes toward donated embryo acceptance

On other hand, continental subgroup analysis revealed that five studies were conducted in Asia, with pooled prevalence of 23.73% (95% CI 13.32–34.13) (*I*^*2*^ = 97.82%, *p* = 0.00), and three studies were conducted in non-Asian countries with pooled prevalence of positive attitudes toward donated embryo acceptance of 47.78% (95% CI 2.24–93.31) (*I*^*2*^ = 99.85%, *p* = 0.00) (Fig. 8).

**Fig. 9:**
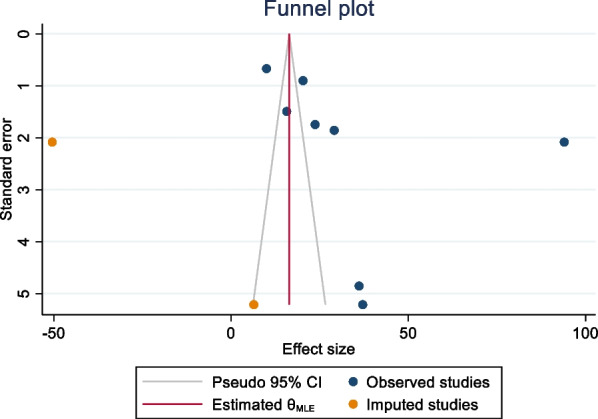
Forest plot showing the global pooled prevalence of positive attitudes toward embryo acceptance per 100 among infertile couples 2022

Publication bias was assessed using funnel plots and objectively verified using Egger’s regression test. The funnel plot appeared asymmetric, although Egger’s regression test (p = 0.5299) did not confirm the asymmetry of the funnel plot (Fig. 9). Finally, the funnel plots appear asymmetrically pin-pointed to the left for the global pooled prevalence of positive attitudes toward donated embryo acceptance.

### Global pooled prevalence of positive attitudes toward donated sperm acceptance

Ten studies with a sample size of 6513 were included in the final analysis, and the individual study sample sizes ranged from 86 to 2007 [19, 22]. The range of positive attitudes toward embryo acceptance was reported from 10% in Japan [13] to 94% in Spain [38].

**Fig. 10:**
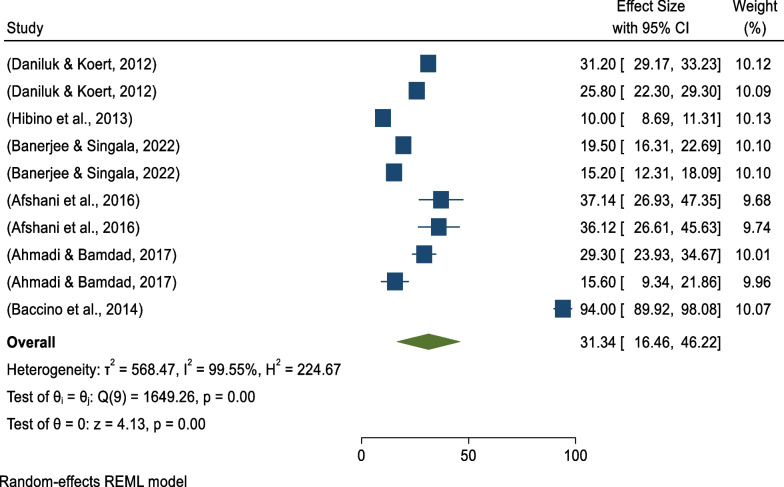
Forest plot showing continental sub-group analysis for pooled prevalence of positive attitudes toward donated sperm acceptance

**Fig. 11:**
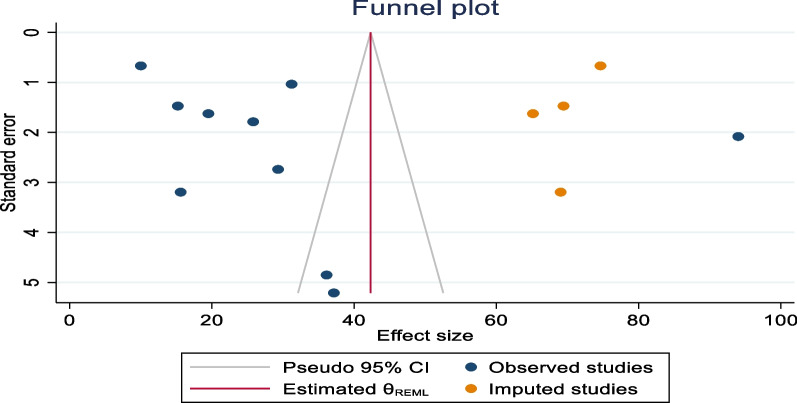
Funnel plot showing the trim-and-fill analysis on global pooled prevalence of positive attitudes toward sperm acceptance

Based on the random-effects model, the global pooled prevalence of positive attitudes toward sperm acceptance was 31.34% (95% CI 16.46%–46.22%) (Fig. 10). There was high heterogeneity among studies (*I*^*2*^ = 99.55%, *p* = 0.00), and based on the trim-and-fill analysis learner estimator imputing four studies to the right (observed 10+ imputed 4 = 14 studies), the global pooled estimate became 42.21% (95% CI 28.22–56.40) after trim-and-fill analysis (Fig. 11).

### Subgroup analysis of positive attitudes toward sperm acceptance

**Fig. 12:**
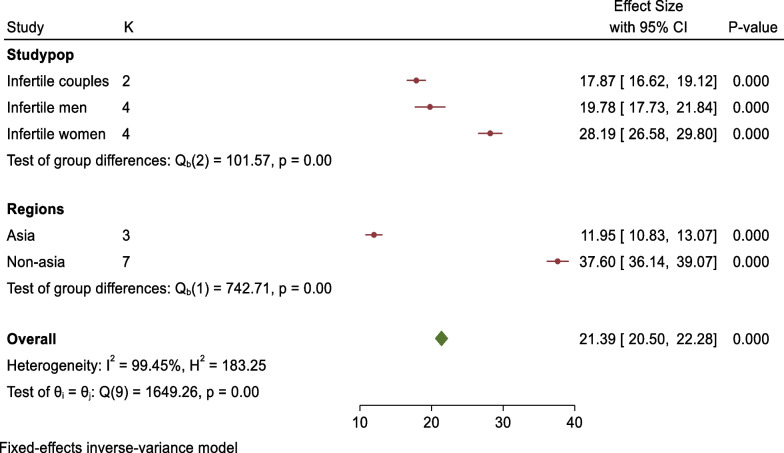
Forest plot showing sub-group analysis for pooled prevalence of positive attitudes toward donated sperm acceptance

According to the category of study participants, subgroup analysis revealed that two studies were conducted on infertile couples at the public level with pooled prevalence of positive attitudes toward sperm acceptance of 17.87 (95% CI 16.62–19.12, p = 0.000), four studies were conducted on infertile men with pooled prevalence of positive attitudes toward sperm acceptance of 19.78 (95% CI 17.73–21.84, p = *0.000*), and four studies were conducted on infertile women with pooled prevalence of positive attitudes toward sperm acceptance of 28.19 (95% CI 26.58–29.80, p = 0.000). Based on the study country, three studies were conducted in Asian countries with pooled prevalence of positive attitudes toward sperm acceptance of 11.95 (95% CI 10.83–13.07, p = 0.000), and seven studies were conducted in non-Asian countries with pooled prevalence of positive attitudes toward sperm acceptance of 37.60 (95%CI 36.14–39.07, p = 0.000) (Fig. 12).

## Discussion

The systematic review and meta-analysis identified a wide range of positive attitudes toward the acceptance of donor eggs, embryos, and sperm. The pooled prevalence of positive attitudes toward the acceptance of donor eggs in this study agrees with the findings of a global report, which stated low ART utilization in Latin America [3] and less willingness to use donated eggs in Canada [7]. In contrast, this finding is lower than that reported in Australia, which reported a relatively high prevalence of accepting the use of Assisted Reproductive Technology [4]. The reason for this difference could be the involvement of Medicare funding, which has raised acceptance of the service over the years.

In the subgroup analysis, positive attitudes toward accepting donor eggs was higher in infertile men than in infertile women. However, the subgroup analysis of studies conducted on the community (public) was homogeneous, and positive attitudes toward acceptance were higher than those of infertile women. This is in line with a report from Canada [7] in which women expressed less willingness to accept. Moreover, the logic that males are usually open to experiencing new things supports this finding.

In the current study, Asian countries had less favorable (positive) attitudes toward accepting donor eggs than did non-Asian countries. This was also realized in previous individual studies, where findings from Asian countries showed less willingness to accept and utilize Assisted Reproductive Technology [3, 16].

The prevalence of positive attitudes toward accepting donor embryos (33.2%) in this study was lower than the prevalence of positive attitudes to accept donor eggs. Some individuals believe that the embryo is not their child if the accepted treatment for infertility can contribute to this variation. This is why people prefer to accept egg and gamete donation [11]. Similar to accepting egg donation, the positive attitudes toward accepting embryo donation were lower in infertile women than in infertile men and couples. This may be due to women becoming less interested in accepting new experiences than men. Again, non-Asian countries have more positive attitudes toward accepting embryo donation than do Asian countries. This is similar to positive attitudes toward accepting egg donation.

Unlike attitudes toward accepting eggs and embryo donation, the prevalence of positive attitudes toward accepting sperm donations was low. Women may not accept another person’s sperm for infertility treatment, as evidenced in Iran, where the community mainly supports in vitro fertilization (using the husband’s sperm and the wife’s egg) [14]. It has also been shown that infertile couples in India prefer to accept eggs and donate embryos for sperm donation [16]. This could be the reason for the variation.

### Limitation

We tried to address the high publication bias that may have affected the true estimates in the current study by using trim-and-fill analysis. However, authors are unsure of whether the high publication bias was due to the presence of substantial heterogeneity among the studies or un-researched/unpublished studies. Finally, this study should be interpreted considering the important limitations of the data available at the time of publication. These need to be considered when interpreting the results of this meta-analysis. Even though this does not necessarily invalidate our conclusions, publication bias and heterogeneity of studies is inevitable no matter how we try to treat them statistically. Authors would like our readers to consider that the studies included in this meta-analysis are heterogeneous.

## Conclusion

The pooled estimate of the prevalence of positive attitudes toward accepting donor eggs (38.63%) was higher than the prevalence of positive attitudes toward accepting donor embryos (33.20%) and donor sperm (31.34%). Infertile men and non-Asian countries have a higher prevalence of positive attitudes toward accepting donor eggs and embryos, whereas non-Asian countries and infertile women a higher prevalence of positive attitudes toward accepting donor sperms. Female acceptance of donor gametes, embryos, or eggs is higher than male acceptance of donor sperm, and females are more amenable to accepting donor eggs than to accepting donor sperm. Assisted reproduction is a common procedure that involves the use of donated sperms, eggs, or embryos. Infertile couples need to understand the medical and obstetric risks associated with donor-assisted conception to improve their attitudes toward donor conception. This systematic review and meta-analysis recommends strengthening counselling for infertile couples’ attitudes toward donor conception and offering support to those with negative attitudes toward donor sperm, eggs, and embryo acceptance. Therefore, regulatory bodies and policymakers should consider the needs of infertile couples, such as access to counselling and services, by modifying their rules and regulations to ensure the availability of minimum standards for the ethical and safe practice of donor conception as a treatment for infertility at the national and international levels. The recommendations outlined in this review are important for ensuring donor-assisted conception based on the potential needs of infertile couples, and for providing opportunities for all parties involved in donor conception to have access to counselling to increase awareness in the community and avail services to satisfy their reproductive rights of self-replacement.

This is the first systematic review and meta-analysis to examine attitudes toward the acceptance of donor eggs, sperm, and embryos as treatments for human infertility globally. There is little literature and no study has been carried out in most countries; therefore, the authors strongly recommend a primary study that explores attitudes toward the acceptance of donor eggs, sperm, and embryos as treatments for human infertility through a comparative study among male and female populations based on a national or subnational level in a country that has never been studied in this area.

### Supplementary Information

**Additional file 1: Appendix S1.** Completed PRISMA 2009 Checklist.

## Data Availability

All data generated or analyzed during this study are included in this article and its supplementary information files.
